# Acetaminophen and pregnancy: common exposure, potential placental impact

**DOI:** 10.1093/jas/skag209

**Published:** 2026-09-02

**Authors:** Brittney-Shania Etuka, Caitlin R Eason, Emma M Golden, Sarkis Christopher Derderian, Clyde J Wright

**Affiliations:** Department of Biological Sciences, University of Denver, Denver, CO 80208, Unites States; Department of Surgery, University of Colorado Anschutz School of Medicine, Aurora, CO 80045, United States; Section of Neonatology, Department of Pediatrics, University of Colorado School of Medicine, Aurora, CO 80045, United States; Department of Surgery, University of Colorado Anschutz School of Medicine, Aurora, CO 80045, United States; Section of Neonatology, Department of Pediatrics, University of Colorado School of Medicine, Aurora, CO 80045, United States

**Keywords:** acetaminophen, paracetamol, placenta, preeclampsia, fetal growth restriction, CYP2E1

## Abstract

Acetaminophen (acetaminophen, APAP), alternatively referred to as paracetamol, is generally accepted as a safe pharmacologic choice to treat pain and fever during pregnancy. This review examines the prevalence and patterns of acetaminophen use during pregnancy and the historical context behind this widespread use. We discuss the emerging and controversial data linking acetaminophen use during pregnancy and adverse effects on the fetus and offspring. Furthermore, we draw on mechanistic evidence from preclinical studies and observational clinical studies that support the hypothesis that in addition to the fetus and newborn, there is a potential impact of acetaminophen on the placenta. We draw on mechanistic data from pre-clinical experiments and observational data from clinical studies to assess the association between APAP exposure and placental cellular injury, preeclampsia, and growth restriction. We conclude that additional research is needed to ensure the most informed pharmacologic management of pain and fever during pregnancy.

## Teaser

Acetaminophen (acetaminophen) is generally accepted as a safe way to treat fever and pain during pregnancy. This review examines the commonality of acetaminophen exposures during pregnancy, the historical context that explains the widespread and growing use of acetaminophen during pregnancy, and the biological plausibility of placental susceptibility to these exposures.

## Introduction

Acetaminophen (chemical abbreviation acetaminophen), alternatively referred to as paracetamol, is one of the most popular over-the-counter antipyretic and analgesic drugs in the world (Lee 2017). In pregnancy, acetaminophen is widely regarded as a safe—and only—over-the-counter therapeutic option for treatment of pain and fever (Fleischman 2025). If left untreated, maternal pain and fever pose significant risk to the fetus and mother. While it is important to acknowledge its important role when therapeutically necessary, acetaminophen is perhaps better characterized as the least harmful therapeutic option in pregnancy. Otherwise stated, an acknowledged therapeutic role when medically indicated does not equate to no or minimal risk to maternal or fetal health.

Emerging studies have identified associations between gestational acetaminophen exposure and a variety of adverse outcomes in offspring, including neurodevelopmental disorders such as autism spectrum disorder (ASD) and attention-deficit/hyperactivity disorder (ADHD), reproductive abnormalities, asthma, and lower intelligence quotient (IQ) scores (Toda 2017; Bauer et al. 2021; Buhrer et al. 2021). While not all evidence points to causality, the consistency of such findings in large cohort studies, sibling-controlled analyses, and preclinical studies suggests that the potential risks may be significant enough to warrant further investigation. Compounding this concern is the observation that the dose–response relationship, timing of exposure, and genetic susceptibility may all modulate the degree of fetal risk (van den Anker and Allegaert 2020).

While considerable focus has been placed on the potential impact of gestational acetaminophen exposure on the offspring, emerging data suggest effects on the placenta and placental function. As we will discuss below, clinical research has linked gestational acetaminophen exposure to preeclampsia and IUGR. Importantly, these observational associations do not imply causation. However, preclinical data demonstrate that acetaminophen can directly affect the placenta. Whether acetaminophen alters placental function or structure through mechanisms such as oxidative stress, inflammation, or disruption of signaling pathways, carries critical implications for fetal health is not definitely known. This becomes particularly concerning when considered in conjunction with the ubiquity of acetaminophen use during pregnancy and its widespread endorsement as a first-line pharmacologic agent.

This review addresses the three following questions: 1) How common is maternal use of acetaminophen, and what are the primary factors driving its use during pregnancy. The objective of the first part of this review is for the reader to be able to identify acetaminophen as one of the most common pharmacologic exposures during pregnancy. 2) What historical developments and regulatory decisions have shaped the prevailing views on acetaminophen’s safety profile during pregnancy? The objective of the second part of the review is for the reader to be able to describe how acetaminophen use during pregnancy was gradually accepted in the absence of preclinical and clinical study demonstrating safety, and how emerging yet controversial data point to a potential risk on the fetus and offspring. 3) Is the placenta, a critical mediator of fetal development, susceptible to injury or dysfunction due to acetaminophen exposure? The objective of the third part of this review is for the reader to recognize that emerging data demonstrate that acetaminophen can impact the placenta, and additional research is warranted. Finally, we consider measures that may inform future study and empower patients and health care practitioners with knowledge to support safe and effective use of acetaminophen during pregnancy.

## Commonality of maternal acetaminophen use during pregnancy in the United States

Acetaminophen’s over-the-counter availability, long-standing reputation as a safe analgesic, and clinical support as the recommended substitute for opioids and non-steroidal anti-inflammatory drugs (NSAIDs), both of which have known risks during pregnancy, account for its widespread use during pregnancy (Black and Hill 2003; Werler et al. 2005; Thiele et al. 2013). Within US cohorts, it has been reported that 50–65% of pregnant women use acetaminophen at least once during their pregnancy, with approximately 3–20% of women reporting use in all three trimesters (Black and Hill 2003; Werler et al. 2005; Servey and Chang 2014; Lee 2017; Arneja et al. 2020; Bandoli et al. 2020; Powers et al. 2023; Guo et al. 2025). The distribution and implications of this timing vary across domestic studies (Bandoli et al. 2020; Woodbury et al. 2024). In the United States, common indications for acetaminophen during pregnancy include chronic pain, headaches, injury, illness, and insomnia (Bandoli et al. 2020; Liew and Ernst 2021). Consistent with these indications, over two-thirds of acetaminophen use is reported as self-directed, without physician oversight, reinforcing concerns about risk communication and informed decision making. US longitudinal data indicate that while most reported use is short-term (<10 consecutive days), 9% of individuals report use for ≥45 d (Bandoli et al. 2020). Increasing duration of acetaminophen (from <10 to >45 d) use was strongly associated with maternal obesity, tobacco use, self-reported depression or anxiety, and concurrent antidepressant use (Bandoli et al. 2020). Additional work, including the use of sibling studies, are necessary to understand the independent influence of these confounders on fetal development in the setting of acetaminophen exposure.

Observational data linking exposures and outcomes are strengthened by demonstrating a dose–response relationship. U.S.-based studies that have evaluated different timepoints and durations of exposure over pregnancy emphasize that short-term use might be relatively benign, various amounts or repeated or extended exposures throughout pregnancy may increase the risk for adverse fetal outcomes (Liew and Ernst 2021; Patel et al. 2022). However, it is important to note that these studies rely on self-reported acetaminophen use, which is prone to recall inaccuracies and limits precise assessment of exposure timing. This is a major limitation when providing acetaminophen use recommendation to pregnant women, as precise data on dose, duration and timing during gestation and over fetal development are lacking.

### Global variation and socioeconomic influences

Prevalence varies substantially from one continent to another, reflecting differences in healthcare practices, public awareness, and trust in medications during pregnancy. Zafeiri et al. (2021) have provided a recent review of the approximately 20 global studies interrogated analgesic use during pregnancy. The vast majority of included studies identify acetaminophen as the most common analgesic used by pregnant women (Zafeiri et al. 2021). Furthermore, these published data note wide variability in the prevalence of acetaminophen usage across countries, with higher rates observed in the United States and Northern/Western Europe and Australia, and relatively lower rates in Eastern Europe, Africa, the Middle East, and South America (Lupattelli et al. 2014; Kristensen et al. 2016; Bandoli et al. 2020; Zafeiri et al. 2021). These differences may stem from variations in over-the-counter availability, physician prescribing patterns, and public awareness campaigns.

Socioeconomic status, level of education, employment status, and whether the pregnancy was planned also influenced patterns of use. For instance, women with lower education or unplanned pregnancies were more likely to report using medications without medical consultation (Lupattelli et al. 2014). Importantly, immigrant women in Northern and Western Europe were significantly less likely to report using medications for chronic or long-term conditions, including acetaminophen (Lupattelli et al. 2014). This suggests disparities in access to care, cultural perceptions of pharmaceutical safety, or communication barriers with healthcare providers.

#### Cultural and commercial influence on maternal acetaminophen use

Cultural norms and commercial interests contribute to the widespread use of acetaminophen during pregnancy. Pharmaceutical trends that value accessibility and convenience contribute to this. Complicating this issue is the fact that acetaminophen, is present in hundreds of combination medications, from sleep aids to flu remedies. Importantly, data demonstrate that ingredient information difficult to interpret; notably, only 31% are aware that Tylenol contains acetaminophen (King et al. 2011).

## History: how did we get here? Common drug, growing recognition of potential risk

Today’s widespread use of acetaminophen during pregnancy evolved gradually, reflecting a historical evolution in medical practice, pharmacological substitution and public health messaging. With this slow evolution—and the absence of safe(r) alternative to treat maternal pain and fever—the assumption and acetaminophen use during pregnancy was safe remained unchallenged until recent decades. To understand how acetaminophen gained this elevated status, and why its safety profile is now under re-evaluation, we must examine both the pharmacological history of analgesics in pregnancy and the shifting scientific and regulatory landscape.

### The origins of acetaminophen

The history of acetaminophen is well documented (Spooner and Harvey 1976; Prescott 2000; Brune et al. 2015). Its discovery was an accident—in 1884 a patient with fever and worms was accidentally given acetanilide instead of naphthalene. This inadvertent treatment with an acetaminophen precursor did nothing for the worms but treated the fever. Complicated by causing methemoglobinemia, this drug was discarded and researchers sought less toxic chemical derivatives. In the early 1900s, the derivative phenacetin was developed. This could be produced at low costs from a waste product of aniline dye production. Simultaneously, aspirin and phenazone entered the market and all three compounds were distributed widely. The search for additional compounds led to the interrogation of the chemically related acetaminophen/paracetamol—however this was deemed not superior to phenacetin and was widely disregarded. It wasn’t until the 1940s that chemists realized that paracetamol was in fact an active metabolite of phenacetin. Importantly, aspirin, phenazone, and phenacetin flooded the market prior to pre-clinical and clinical studies demonstrating safety. Clinical reports began to accumulate linking phenacetin to interstitial nephritis, and by the 1950s, acetaminophen use began to increase. Thus, an untested drug remained on the market and available for over-the-counter consumption for nearly 50 yr prior to the observation linking exposure and toxicity that led to the removal of the drug from the market. Unfortunately, the replacement was similarly untested prior to widespread use.

### The decline of aspirin and the rise of acetaminophen

Acetaminophen use was not complicated by causing interstitial nephritis and therefore use increased throughout the 1950s and 1960s. Acetaminophen had been available in the United States without a prescription since 1955 and was branded as a “gentle” alternative to older drugs such as aspirin (Spooner and Harvey 1976). It was during this time that significant use among pregnant women and children began. Of note, the first report of acetaminophen causing liver toxicity did not appear until 1966 and mechanisms underlying toxicity not discovered until the early 1970s (Hinson et al. 2025)—a reminder of just how much was not known about this drug prior to widespread and over-the-counter use (Prescott 2000; Lee 2017).

Use in children and pregnant women further increased after the link between aspirin taken during a viral illness and Reye’s syndrome—a rare condition causing liver and neurologic injury—was reported in the 1980s (Brune et al. 2015; McCrae et al. 2018). It is important to recognize that the favorable sentiment in the medical community toward acetaminophen was based in the lack of better alternatives rather than any safety profile (Spooner and Harvey 1976; Rudolph 1981; Niederhoff and Zahradnik 1983). Importantly, no pre-clinical or clinical studies were performed to test whether acetaminophen use was safe during pregnancy prior to its widespread use. Of note, this period was marked by a lack of rigorous drug testing in pregnant populations and even exclusion of pregnant women from most randomized controlled trials due to ethical concerns and liability risks (Black and Hill 2003). These factors allowed acetaminophen to exist in obstetric care without robust interrogation of safety.

### The potential consequences of acetaminophen exposures on fetal/neonatal outcomes: emerging questions, consensus statements, and responses

In the absence of preclinical and clinical studies evaluating the effect of in utero acetaminophen exposures, reports of potential harm were slow to develop. Most safety data for medications used in pregnancy, including acetaminophen, were gathered from post-marketing surveillance and observational studies, and reported only that acetaminophen use during pregnancy did not increase the incidence of birth defects or stillbirth (IARC Working Group 1990). It was not until the 2010s that a series of high-profile studies began to associate prenatal acetaminophen exposure with neurodevelopmental disorders such as ADHD and autism spectrum disorder (Liew and Ernst 2021). Additional studies linked prenatal acetaminophen exposures to childhood morbidities including atopic and reproductive outcomes (Liew and Ernst 2021; Patel et al. 2022). Although the validity of these associations has been debated, they have prompted re-examination of earlier assumptions. It has been observed that earlier studies had failed to account for exposure timing, dose, and maternal comorbidities, which are now considered critical confounders (Liew and Ernst 2021). Importantly, Liew’s review highlighted how many of the earlier studies were limited by insufficient statistical power or lacked long-term follow-up, making it difficult to detect subtle developmental sequelae.

The high-profile publication “Paracetamol use during pregnancy–a call for precautionary action” was published in 2021. Labeled as a “consensus statement,” Bauer and colleagues deliver specific recommendations regarding acetaminophen use during pregnancy. Specifically, they state: “pregnant women should be cautioned at the beginning of pregnancy to: forego acetaminophen unless its use is medically indicated; consult with a physician or pharmacist if they are uncertain whether use is indicated and before using on a long-term basis; and minimize exposure by using the lowest effective dose for the shortest possible time” (Bauer et al. 2021). The authors base these recommendations on the accumulating experimental and epidemiologic data linking fetal acetaminophen exposure and increased risk of neurodevelopmental, reproductive, and urogenital disorders. Central to these recommendations is the assessment that experimental data are lacking to make definitive assessments of safety. The authors also acknowledge the limitation of the existing clinical and epidemiological data but argue that potential harms will increase unchecked in the absence of precautionary action.

This publication drew a response from multiple international clinical bodies. Less than a week after the Bauer publication became available online, the American College of Obstetricians and Gynecologists issued a statement arguing that no change should be made in current practice, and that women should not be “frightened away from the many benefits of acetaminophen” (American College of Obstetrics and Gynecology 2021). This statement re-affirms the 2017 assessment of the Society for Maternal Fetal Medicine “that acetaminophen be considered a reasonable and appropriate medication choice for the treatment of pain and/or fever during pregnancy” (Society for Maternal-Fetal Medicine Publications Committee. Electronic address 2017).

In the following months, both the Society of Obstetricians and Gynecologists of Canada and the European Network of Teratology Information Services responded (Hutson et al. 2021; The European Network of Teratology Information Services 2021). The Society of Obstetricians and Gynecologists of Canada argued for no change in clinical practice and recommends “acetaminophen as a first-line therapeutic option for fever and pain in pregnancy” (Hutson et al. 2021). They also argued that the Bauer publication, lacking the support of an organized governing body, is less of a consensus statement and closer to an opinion piece. The European Network of Teratology Information Services statement echoed this concern, noting the supporting data was “weak, inconsistent and to a large extent fundamentally flawed” (The European Network of Teratology Information Services 2021). Furthermore, they noted “that the media headlines and social media reactions were predictable, and European Network of Teratology Information Services believed that this paper and the ensuing reaction would promote unwarranted uncertainty, fear, and guilt among pregnant women. It would also likely result in use of less safe alternatives during pregnancy” (The European Network of Teratology Information Services 2021).

Recent statements from the White House have brought additional attention to the use of acetaminophen during pregnancy (House 2025). The FDA released a statement emphasizing that association between exposure and outcome does not indicate causation, and the acknowledged conflicting reports on a number of the associations noted (Administration 2025). In response, the American College of Gynecology has reaffirmed the role of acetaminophen as the analgesic and antipyretic of choice during pregnancy (ACOG 2025).

It is important to note that chasm in the narrative regarding acetaminophen use during pregnancy. The White House statement sounds definitive regarding the association between exposure and outcome. Statements by the American College of Obstetrics and Gynecology, Society of Obstetricians and Gynecologists of Canada or European Network of Teratology Information Services do not identify the need for further research. However, while the body of literature interrogating the potential effect of acetaminophen on the developing fetus continues to grow, whether the placenta is susceptible to acetaminophen-induced toxicity remains to be fully tested. This knowledge gap has been identified in recent reviews. Specifically, while prenatal acetaminophen exposure has also been linked to neurodevelopmental, atopic, and reproductive outcomes, whether these associations may result from a direct effect on the placental and subsequent injury and dysfunction has not been investigated (Patel et al. 2022). Here we highlight findings in studies interrogating the effect of acetaminophen on the placenta, identify areas for additional study.

## The potential effect of acetaminophen on the placenta: pre-clinical and clinical data

The placenta serves as the interface for the maternal–fetal dyad. It facilitates nutrient, gas, and waste exchange while also functioning as a selective barrier against toxic substrates. Below, we highlight studies demonstrating that maternal ingestion of acetaminophen results in placental exposures. Furthermore, the placenta expresses Cyp2e1, the enzyme responsible for the production of acetaminophen-derived toxic metabolites. We review studies linking Cyp2e1 expression to potential placental toxicity. Finally, we discuss clinical studies demonstrating a potential link between maternal acetaminophen exposure and placental dysfunction. Through this review, we hope to highlight the need to further characterize how acetaminophen affects the placenta.

### Placental exposure to acetaminophen

Preclinical data demonstrate rapid and robust placental transfer of acetaminophen following maternal exposure. In pregnant mice, maternal administration of acetaminophen results in comparable concentrations and rates of degradation in maternal blood, maternal liver, placenta, and fetal liver (Lambert and Thorgeirsson 1976). In pregnant sheep, acetaminophen is transferred bidirectionally via passive diffusion with similar maternal and fetal clearance rates (Wang et al. 1986).

Similar results are found in humans exposed to acetaminophen. Acetaminophen in the maternal bloodstream rapidly diffuses across placental tissues, entering the fetal circulation within minutes of ingestion (Levy et al. 1975; Naga Rani et al. 1989; Nitsche et al. 2017; Guo et al. 2025). In a study of 34 women given a 1-g dose of acetaminophen before scheduled cesarean delivery, maternal venous, and cord blood concentrations showed nearly identical pharmacokinetics, with transfer ratios approaching 90% (Nitsche et al. 2017). A unique case of a woman at 36 wk who delivered 6 h after an acetaminophen overdose demonstrated comparable maternal and neonatal plasma concentrations suggests free placental passage (Roberts et al. 1984). Using an ex vivo dual-perfusion system of full-term human placentas, constant fetal-to-maternal acetaminophen ratios were observed within 1 h (Weigand et al. 1984). Another ex vivo study showed a placental transfer rate of 45% through passive diffusion (Conings et al. 2019). Collectively, these findings confirm that across multiple species, maternal acetaminophen ingestion reliably results in fetal and placental exposure.

### Mechanisms underlying acetaminophen-induced injury: the role of Cyp2e1

**Figure 1:**
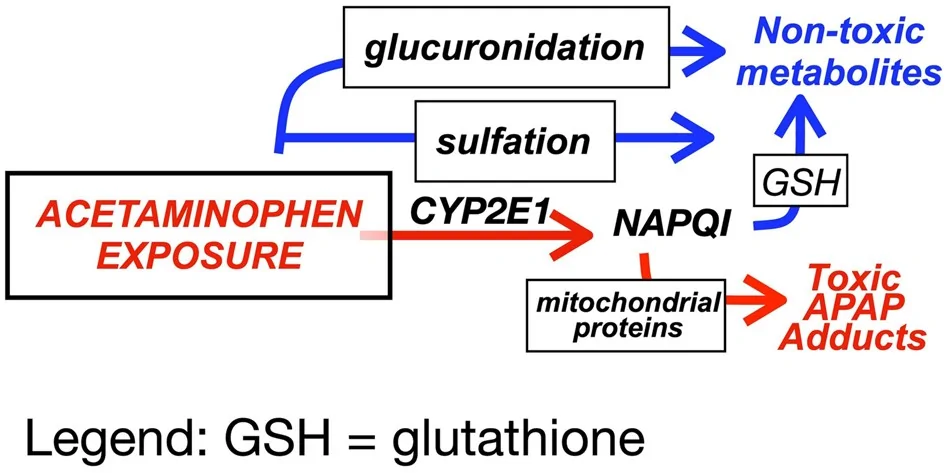
Pathways of acetaminophen metabolism. GSH = glutathione.

The mechanism of acetaminophen-induced cellular toxicity is well established (Figure 1) (Jaeschke and Ramachandran 2024). In adults, hepatic metabolism primarily occurs through glucuronidation and sulfation. When these pathways are saturated, CYP2E1 is engaged and converts acetaminophen to the reactive metabolite *N*-acetyl-p-benzoquinone imine (NAPQI). Under normal conditions, NAPQI is detoxified through conjugation with glutathione (GSH). However, when glucuronidation and sulfation are overwhelmed and glutathione stores depleted, NAPQI binds to mitochondrial proteins, leading to metabolic dysfunction and cell death. Consequently, toxicity is closely linked to cell type–specific expression of CYP2E1.

### CYP2E1 is expressed in the placenta

**Figure 2:**
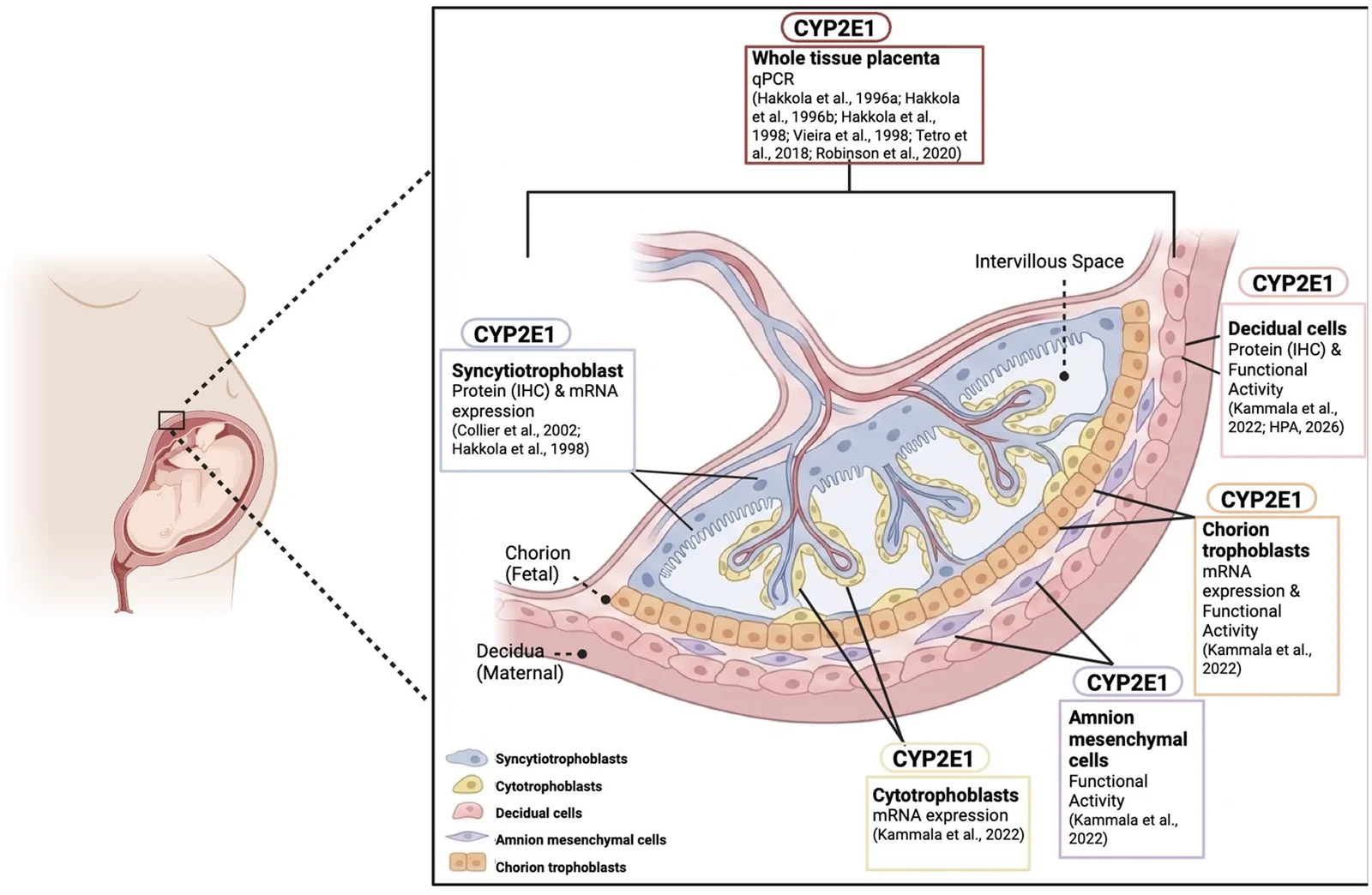
Cell type specific expression of CYP2E1 in the human placenta. Placental anatomy and locations of documented CYP2E1 expression, with references noted. Made with BioRender.

Cell type-specific expression of CYP2E1 dictates acetaminophen induced toxicity. Multiple studies have documented placental CYP2E1 expression (Figure 2). Pre-clinical studies have detected CYP2E1 expression in rat and murine placenta (Ejiri et al. 2005; Wang et al. 2009; Martin-Estal et al. 2022). Therefore, whether the human placenta expresses CYP2E1, and is thereby potentially susceptible to acetaminophen toxicity is a key consideration. Importantly, CYP2E1 placental expression has been studied and extensively reviewed (Hakkola et al. 1998; Myllynen et al. 2009; Tetro et al. 2018). CYP2E1 has been detected by qPCR across all three trimesters (Hakkola et al. 1996a, 1996b, 1998; Vieira et al. 1998; Tetro et al. 2018; Robinson et al. 2020). Immunohistochemistry of 25 first-trimester placentas localized CYP2E1 protein predominantly to the syncytium (Collier et al. 2002), a finding confirmed in decidual and trophoblastic cells by the Human Protein Atlas (2026). Consistent with protein expression, a detailed analysis of five term placentas demonstrated functional CYP2E1 activity in amnion mesenchymal cells, chorion trophoblasts, and decidual cells using a fluorophore-based enzymatic assay. CYP2E1 mRNA was also expressed across trophoblasts and other placental cell types, though expression levels varied (Kammala et al. 2022). Expression later in gestation appears more variable, with some evidence suggesting environmental influences including smoking, polybrominated diphenyl ethers and organochlorines (Vieira et al. 1998; Robinson et al. 2020).

### Placental *Cyp2e1* expression is inducible by environmental stressors

Environmental exposures, in particular ethanol and nicotine, can upregulate placental *Cyp2e1* expression. Regulation occurs at transcriptional, post-transcriptional, translational, and post-translational levels (Hakkola et al. 1998; Novak and Woodcroft 2000). In rodents, ethanol and nicotine exposure increase hepatic and placental *Cyp2e1* (Wang et al. 2009). However, small studies using human samples fail to demonstrate an association between smoking and placental CYP2E1 expression (Collier et al. 2002; Czekaj et al. 2005). In pregnant mice, ethanol consumption induces maternal hepatic expression of *Cyp2e1* and increases expression in the placental junctional zone (Martin-Estal et al. 2022). In humans, term placentas from women with heavy ethanol use showed CYP2E1 protein expression that correlated with fetal signs of Fetal Alcohol Syndrome (Rasheed et al. 1997). A study of 50 s-trimester placentas also demonstrated CYP2E1 induction following PBDE exposure (Zota et al. 2018). Regulation by xenobiotics may involve epigenetic mechanisms (eg methylation) or protein stabilization, both of which can enhance toxic metabolite formation and oxidative stress (Hakkola et al. 1998; Novak and Woodcroft 2000).

### Preclinical data suggest placental injury from acetaminophen exposure

As CYP2E1 is a known metabolizer of acetaminophen and is expressed in the placenta, several studies have studied the effect of exposures on placental cells. We will present both in vitro and in vivo work, focusing first on the impact of exposures on specific cells, and then on placental function and the potential impact of impaired placental function on the fetus and offspring (Table 1).

**Table 1:** In vitro and in vivo studies testing the effect of acetaminophen on the placenta.

In vitro	Cell type	Organism/tissue	Dose duration	Cyp2e1 expression	Outcome	Refs.
	JAr	Human trophoblast	20 μM to 40 mM 48 h	Negligible	None noted	Blazquez et al. (2014)
	JEG-3	Human trophoblast	20 μM to 40 mM 48 h	Negligible	Decreased BCRP (breast cancer resistance protein) Nrf2 activation + target gene expression and ROS generation	Blazquez et al. (2014)
	BeWo	Human trophoblast	20 μM to 40 mM 48 h	Negligible	None noted	Blazquez et al. (2014)
	JEG-3	Human trophoblast	1 to 5 mM 24–48 h	Not stated	Decreased cell viability at highest exposure Alterations in expression of multiple genes related to hormone regulation	Addo et al. (2021)
	HTR-8/SVneo	Human trophoblast	10 μM or 1 mM	Not stated	No change in cell viability Decrease in angiogenic and vascular remodeling gene expression Increase in cell migration Inhibition of trophoblast invasion + inhibition pro-invasive gene expression	Burman et al. (2021)

**In vivo**	**Organism**	**Timing**	**Dose**	**Cyp2e1 expression**	**Outcome**	**Refs.**

	Rat	E18 × 3 d	400 mg/kg IP	Not stated	Decreased BCRP (breast cancer resistance protein) higher bile acid accumulation in the placenta during maternal cholestasis + glutathione consumption	Blazquez et al. (2014)
	Rat	E8–19	125/250 mg/kg IP	Not stated	No effect on birth weight	Lubawy and Garrett (1977)
	Rat	E8–14	3.5/35/350 mg/kg IP	Not stated	Dose-dependent decrease in placental and fetal body weight	Burdan (2003)
	Mice	E12.5	50/250 mg/kg IP	Not stated	Dose-dependent decrease in placental and fetal body weight at E13.5 and E16.5	Karimi et al. (2015)
	Mice	E12.5	50/250 mg/kg IP	Not stated	Dose-dependent decrease in placental and fetal body weight at E13.5 and E16.5	Thiele et al. (2015)
	Mice	Continuously	0/357/715/1,430 mg/kg PO	Not stated	No difference in birth weight	Reel et al. (1992)
	Mice	E11.5–13.5	15/50/150 mg/kg PO	Not stated	Dose-dependent decrease in fetal weight at E14.5, no impact on placental weight Impaired placental metabolomics Impaired placental angiogenesis	Dai et al. (2024)
	Rat	E15–19 E19	15 mg/kg BID 15 mg/kg	Not stated	Placental RNAseq demonstrated alteration in the expression of acute phase and immune related genes	Koehn (2020)

Blazquez and colleagues used human trophoblast cell lines to evaluate the effect of acetaminophen on placental barrier function in the context of maternal cholestasis (Blazquez et al. 2014). Although they reported Cyp2e1 expression as “negligible as compared with human liver (data not shown),” they did find evidence of acetaminophen induced ROS production and Nrf2 activation and expression of target genes in the JEG-3 cell line. Importantly, in vivo exposures resulted in impaired placental barrier function when assessed using both an isolated perfused placental model and interrogation of maternal and fetal tissues for bile acid content. Furthermore, acetaminophen exposure resulted in placental glutathione consumption, a finding consistent with detoxification of CYP2E1 derived acetaminophen metabolites. Additional studies using the human trophoblast JEG-3 cell line demonstrated decreased cell viability at the higher range of dose-response experiments (5 mM) and alterations in multiple genes related to hormone regulation (Addo et al. 2021). In studies using a different human trophoblast cell line (HTR-8/SVneo), acetaminophen exposures that did not induce cell death were shown to inhibit trophoblast invasion and decrease the expression of genes related to angiogenesis, vascular remodeling and invasion (Burman et al. 2021). Interestingly, authors were able to identify genes impacted by acetaminophen that overlapped with genes altered in placentas from preeclamptic subjects. The authors concluded that these findings were consistent with acetaminophen interfering with critical trophoblast functions.

A number of in vivo studies have investigated the impact of maternal acetaminophen exposures on fetal weight. The earliest rat study in 1977 reported no significant effect on offspring weight (Lubawy and Garrett 1977). Later studies, however, demonstrated reductions in placental and fetal weight after hepatotoxic acetaminophen (350 mg/kg) exposures (Burdan 2003). In mice, both non-hepatotoxic (50 mg/kg) and hepatotoxic exposures (250 mg/kg) administered at E12.5 decrease fetal weight at gestational days 13.5 and 16.5 (Karimi et al. 2015; Thiele et al. 2015). Similarly, acetaminophen 150 mg/kg PO administered on E11.5–13.5 decreased fetal weight at E14.5 and disrupted placental angiogenesis (Dai et al. 2024). High-dose exposure was also associated with reduced birth weight at term (Karimi et al. 2015). Even postnatally, mice exposed to prenatal acetaminophen showed poor weight gain up to 74 d and reduced fecundity, despite no initial birth weight difference (Reel et al. 1992). Recent data may help elucidate mechanisms as both acute (15 mg/kg IP × 1 at E19) and chronic exposure (15 mg/kg IP BID E15–19) to acetaminophen induce robust expression of immune related genes in rat placenta (Koehn et al. 2020).

### Clinical data associate acetaminophen exposure and placental manifestations

Preclinical findings of acetaminophen-induced placental dysfunction may translate to human disease, including preeclampsia and impaired growth. Two large studies (combined ∼ 66,000 subjects) found associations between prenatal acetaminophen use and preeclampsia, though they did not report on birth weight (Rebordosa et al. 2010; von Hellens et al. 2021). Another study identified that acetaminophen increased preeclampsia risk among women with autoimmune disorders (Killion et al. 2022). These associations align with cellular effects observed in vitro (Burman et al. 2021).

Although reports are inconsistent, acetaminophen exposure has been linked to impaired fetal growth. Interestingly, the Canadian birth cohort reported that preconception acetaminophen use was associated with impaired growth, with at least weekly use in the 3 mo before conception doubling the risk of LBW and nearly doubling the risk of SGA (Arneja et al. 2020). In a cohort of >150,000 mothers, acetaminophen use was associated with increased risk of low birth weight (LBW) (Zafeiri et al. 2022). Acetaminophen detected in meconium has been associated with decreased birth weight (Baker et al. 2022). Among ∼100,000 mothers with preeclampsia, acetaminophen use was linked to preterm birth but not small for gestational age (SGA) (Rebordosa et al. 2009). Several other studies found no association with SGA, fetal growth, or birth weight (Thulstrup et al. 1999; Rebordosa et al. 2009; Smarr et al. 2019; de Castro et al. 2022; Delker et al. 2023; Xu et al. 2024), a finding supported by a systematic review and meta-analysis (Castro et al. 2022). The reasons underlying these conflicting data are not known, but likely due to the limitations of observational studies. Knowledge of the exposure, including dose and timing, is imprecise. Given the multifactorial etiology of the outcomes of interest, large samples are necessary with appropriate statistical approaches to adjust for appropriate confounders. However, given these observations, continued pre-clinical, and clinical investigation is needed.

## Future directions

The widespread use of acetaminophen during pregnancy, together with the growing number of pre-clinical and clinical reports demonstrating a potential negative impact on the placenta supports the need for further research to empower the safest approach to treating pain and fever in pregnancy. Furthermore, real concerns exist regarding the real-life implications on clinical practice in the absence of a definitive relationship between acetaminophen exposure and adverse outcomes. As suggested by the SOGC and ENTIS statement, these include deleterious impacts on the fetus from untreated pain and fever, exposure to untested alternatives, and maternal guilt and uncertainty. As a result, we find ourselves in a liminal period: one where acetaminophen continues to be widely recommended, but under growing scientific scrutiny.

The history of acetaminophen in pregnancy is a case study in the difficulty of performing, objectively interpreting and acting on the results of scientific inquiry that challenge longstanding clinical norms. As new findings emerge, medical institutions and governing bodies face the challenge of reconciling legacy practice with precaution with practicality.

### Limitations and gaps in the literature

Despite the mounting evidence, several limitations remain. Most human studies rely on observational designs, which are vulnerable to confounding and recall bias. Many studies also use self-reported medication use without validating exposure levels or exact timing. Moreover, direct study of placental structure and function in humans is ethically and practically limited, making it difficult to prove causality or delineate precise mechanisms. Animal studies, while illuminating, may not fully translate to human placental physiology, given interspecies differences in structure, hormone production, and metabolic pathways. For example, rodents have a hemotrichorial placenta, which has three layers of cells separating maternal and fetal blood, while humans have a hemochorial placenta, where maternal blood comes into direct contact with the fetal chorion (Aguilera et al. 2022). This structural difference affects how substances are transferred between the mother and fetus.

Despite these limitations, the existing literature strongly suggests that the placenta is not a passive channel, but rather an active and vulnerable target of acetaminophen exposure. The combined effects of oxidative stress, hormonal disruption, immune interference, and metabolic saturation point toward a multi-pronged mechanism of placental injury that could help explain the associations between maternal acetaminophen use and various adverse fetal outcomes.

### Future research directions

Given the limitations of the existing literature, including reliance on self-report data, observational designs, and heterogeneity in exposure classification, future research must prioritize both methodological rigor and mechanistic clarity. Below are key recommendations for future study.

#### Mechanistic pre-clinical placental studies

There is a clear need for pre-clinical research that investigates how acetaminophen affects placental biology. Ex vivo placental perfusion models, transcriptomic and epigenetic analyses of placental tissue, and in vitro studies using trophoblast cell lines may offer insights into oxidative damage, hormonal interference, and transport disruptions. Furthermore, alternative mechanisms of injury independent of CYP2E1 must be interrogated. In addition to CYP2E1-derived oxidative stress, acetaminophen can disrupt placental endocrine, immune, and redox systems, altering downstream developmental pathways (van den Anker and Allegaert 2020; Liew and Ernst 2021). The placenta’s immune role is critical for maternal–fetal tolerance; disruption may increase susceptibility to inflammation and placental insufficiency. Buhrer et al. (2021) further demonstrated that acetaminophen alters expression of cytokines, matrix metalloproteinases, and angiogenic factors essential for vascular development and remodeling. These mechanisms remain largely unexplored.

#### Longitudinal cohort studies

High-quality, prospective birth cohorts with biomarker-validated exposure measurements (eg cord blood, meconium, and maternal serum) are essential to strengthening causal inference. These should track outcomes from birth through adolescence to assess developmental trajectories. Large scale sibling studies could help interrogate the role of confounders.

#### Trimester-specific exposure analysis

Timing matters. Future studies should disaggregate results by gestational age at first exposure, cumulative exposure over time, and critical windows of fetal vulnerability, particularly during neural development and placental remodeling.

#### Dose–response characterization

Most studies categorize acetaminophen exposure as a binary (yes/no) variable. To advance clinical guidelines, researchers must identify specific dosage thresholds that predict risk, especially for repeated and prolonged use.

#### Differential risk by maternal health status

Inflammation, anxiety, and chronic pain are among the conditions that are associated with increased use of acetaminophen and may also have an impact on fetal outcomes. In order to determine whether acetaminophen increases risk beyond these baseline factors, future research should more thoroughly account for these conditions.

#### Health communication and labeling studies

Investigations into how information about acetaminophen safety is conveyed, through pharmacy counseling, clinician discussions, or drug labeling, are important to shape public health campaigns. Research into how these messages influence behavior will help guide more effective interventions.

## Conclusion

Acetaminophen (acetaminophen) has long been considered the analgesic and antipyretic of choice during pregnancy, favored for its relative safety compared to alternatives such as nonsteroidal anti-inflammatory drugs (NSAIDs) or opioids. However, emerging evidence challenges the blanket classification of acetaminophen as benign, particularly during critical windows of fetal development.

The placenta is central to this discussion. Once considered a neutral barrier, it is increasingly recognized as a dynamic and sensitive organ that not only mediates maternal-fetal exchange but also reacts to environmental stressors, pharmaceuticals, and metabolic disruption. Knowing that acetaminophen reaches the fetal environment within minutes of ingestion, and may alter placental structure and signaling, adds biological plausibility to epidemiological findings linking prenatal acetaminophen exposure with adverse birth outcomes (such as low birthweight and small-for-gestational-age infants), neurobehavioral deficits, and altered reproductive development. Clinical guidelines have not changed much in spite of these worries. acetaminophen use during pregnancy is still approved by the European Medicine Agency and the U.S. FDA, albeit with the general disclaimer that it should be used “as needed, at the lowest effective dose, and for the shortest duration possible.” However, these warnings are rarely highlighted in clinical consultations and are frequently missing from over-the-counter labeling. Pregnant women who have less access to prenatal care, are less health literate, or are less aware of the risks associated with medications are disproportionately affected by this mismatch between what is known and what is communicated. Together with improved public health education and more lucid pharmaceutical labeling, additional hypothesis driven preclinical and clinical investigation is needed to close these gaps.

## Abbreviations

ADHD: attention-deficit/hyperactivity disorder
CYP2E1: Cytochrome P450, family 2, subfamily e, polypeptide 1
FDA: Food and Drug Administration
GSH: glutathione
IQ: intelligence quotient
NAPQI: *N*-acetyl-p-benzoquinone imine
NRF2: nuclear factor erythroid 2-related factor 2
NSAIDs: non-steroidal anti-inflammatory drugs
